# Embryonic zebrafish xenograft assay of human cancer metastasis

**DOI:** 10.12688/f1000research.16659.2

**Published:** 2018-12-20

**Authors:** David Hill, Lanpeng Chen, Ewe Snaar-Jagalska, Bill Chaudhry

**Affiliations:** 1 Institute of Cellular Medicine, Newcastle University, UK, Newcastle upon Tyne, NE2 4HH, UK; 2 Institute of Genetic Medicine, Newcastle University, UK, Newcastle upon Tyne, NE1 3BZ, UK; 3 Leiden University, Leiden, The Netherlands

**Keywords:** Zebrafish embryo, xenograft, cancer, melanoma, prostate cancer, metastasis, replacement

## Abstract

Cancer metastasis is the most important prognostic factor determining patient survival, but currently there are very few drugs or therapies that specifically inhibit the invasion and metastasis of cancer cells. Currently, human cancer metastasis is largely studied using transgenic and immunocompromised mouse xenograft models, which are useful for analysing end-point tumour growth but are unable to accurately and reliably monitor *in vivo* invasion, intravasation, extravasation or secondary tumour formation of human cancer cells. Furthermore, limits in our ability to accurately monitor early stages of tumour growth and detect micro-metastases likely results in pain and suffering to the mice used for cancer xenograft experiments. Zebrafish ( *Danio rerio*) embryos, however, offer many advantages as a model system for studying the complex, multi-step processes involved during cancer metastasis. This article describes a detailed method for the analysis of human cancer cell invasion and metastasis in zebrafish embryos before they reach protected status at 5 days post fertilisation. Results demonstrate that human cancer cells actively invade within a zebrafish microenvironment, and form metastatic tumours at secondary tissue sites, suggesting that the mechanisms involved during the different stages of metastasis are conserved between humans and zebrafish, supporting the use of zebrafish embryos as a viable model of human cancer metastasis. We suggest that the embryonic zebrafish xenograft model of human cancer is a tractable laboratory model that can be used to understand cancer biology, and as a direct replacement of mice for the analysis of drugs that target cancer invasion and metastasis.

Research highlights **Scientific benefit(s):**

- Optimal xenotransplantation can be performed in zebrafish embryos at 48 hpf, allowing for a 72-hour period to model key stages of metastatic behaviour.
- Metastatic processes can be visualised at the single cell level.

**3Rs benefit(s):**

- Zebrafish embryos can be used to replace mouse xenograft models in early cancer metastasis research.

**Practical benefit(s):**

- Only a small number of cancer cells (100–200 cells per fish) are required.
- Use of fluorescent cell markers in conjunction with transgenic zebrafish lines allows for host cells to be distinguished from human cancer cells in real-time/ *in situ*, avoiding the need for post-mortem immunocytochemistry.
- The zebrafish embryo assay is higher-throughput than mouse xenograft models.

**Current applications:**

- Studying tumour invasion and metastatic dissemination of different human cancer cell lines using time-lapse microscopy.
- Studying the invasion of human cancer cells into zebrafish blood vessels and in the formation of secondary tumours.

**Potential applications:**

- Studying the heterogeneity of tumours and cooperation of different cancer cells from patient-derived tumours.
- Studying the remodelling of the extracellular matrix during tumour invasion.
- As a screening assay to identify new agents/drugs that reduce the metastatic behaviour of cancer cells.

## Introduction

Metastasis is a clinical term describing the spread of tumour cells from a primary location to distant sites. It is suggested that more than 90% of deaths from cancer are not caused by the primary tumour but by the direct effects of metastatic deposits and from the metabolic burden of a rapidly growing tumour cell mass ( Jemal *et al.*, 2011). Traditionally an orderly cascade of cellular behaviours was presumed to underlie the progression from a well circumscribed and localised tumour growth to distant spread, based on initial local invasion, entry into the vascular or lymphatic system, survival in those fluid channels followed by extravasation and colonisation in a distal site ( Massagué *et al.*, 2017). However, this orderly progression is not borne out by current research and the mechanisms of metastatic spread remain controversial. The role of epithelial to mesenchymal transformation (EMT) is unclear and plasticity of cells that metastasise and their relationship to the primary tumour cells, e.g. stem cells, remains the object of current research ( Pandya *et al.*, 2017). Furthermore, cancers do not appear to disseminate randomly, but exhibit tropism for specific organs, especially lung, liver and bone ( Tarin, 2011). This observation, made over 100 years ago by Steven Paget, led to the "seed and soil" hypothesis which remains unproven. In clinical practice, surgical resection or local treatment of primary tumours is effective, but metastases remain difficult to treat. This is particularly evident for melanoma, where localised and slow-growing metastatic deposits can appear long after apparent cure ( Gershenwald & Scolyer, 2018). Similarly, in prostatic cancer the primary site is rarely a clinical problem in comparison to the pain and pathological fractures from osteolytic vertebral deposits ( Akakura *et al.*, 1996).

Understanding the multi-step processes that regulate cancer metastasis will likely result in new therapeutics to benefit patients with a wide range of cancers at different stages of progression. Although *in vitro* systems, e.g. the artificial skin model for melanoma ( Hill *et al.*, 2015) can be highly effective for studying primary tumour behaviour, connected organ systems are needed to understand metastasis. The mouse has traditionally been used as a pre-clinical model organism to study cancer under the rationale that they are a mammalian species, with the same organ systems as humans. Although genetically modified animals do spontaneously develop tumours, the introduction of human tumour cells into other species, xenografting, is a vital pre-clinical tool that enables researchers to study tumour metastasis and evaluate drug responses ( van Marion *et al.*, 2016). Xenografts provide greater experimental control and can provide a direct translational link to the patient, particularly when the developmental origin of cancer remains unknown. However within a mouse, metastatic spread from xenografts often occurs late, well after the primary deposit has become distressing to the animal, and further pain can also result from the aggressive invasive nature of the metastases ( Gómez-Cuadrado *et al.*, 2017). Highly metastatic cell lines are often used to accelerate the development of metastatic tumours, but these may not reflect normal metastasis, and therefore several different lines must be used, requiring many more animals ( Cruz-Munoz *et al.*, 2008). It is sometimes possible to surgically remove the primary tumour prior to analysis of metastatic dissemination ( Srivastava *et al.*, 2014); however, this is often associated with excessive tissue damage requiring prolonged post-operative analgesia. Direct injection of cancer cells into the tail vein ( Elkin & Vlodavsky, 2001; Minn *et al.*, 2005), heart ( Kang *et al.*, 2003), illiac artery ( Bos *et al.*, 2009; Wang *et al.*, 2015), spleen ( Morikawa *et al.*, 1988), peritoneum ( Chu *et al.*, 2015) or tibia ( Fisher *et al.*, 2002) have all been used to model local metastatic behaviours, but the mouse model is limited since metastasising single cells cannot be tracked and only relatively large metastatic growths can be detected, precluding study of the earliest metastatic events. Furthermore, mouse models have also had limited success when predicting anti-cancer drug efficacy in human trials ( Day *et al.*, 2015; Kersten *et al.*, 2017).

The zebrafish is a tropical bony fish which for over 30 years has been increasingly used in developmental biology and human disease modelling as it contains almost all human organ systems except lungs ( Penberthy *et al.*, 2002). The zebrafish genome has been sequenced and there is a high degree of conserved genes and genetic signalling pathways compared to humans ( Howe *et al.*, 2013). Importantly for the study of cancer metastasis, embryos are completely transparent, facilitating imaging at single cell level within developing organs whilst also imaging the entire animal. Furthermore, the majority of studies can be carried on early-stage embryos before they are capable of independent feeding, which for the zebrafish is widely considered to be 5 days post fertilisation (dpf), and protected under the Animals (Scientific Procedures) Act (ASPA) and EU Directive (2010/63/EU). The extra-uterine development of hundreds of eggs also permits a greater number of studies in genetically identical organisms. Since the first reported xenotransplantation of human cells into zebrafish ( Lee *et al.*, 2005), many laboratories have shown that zebrafish embryos are useful for the study of other facets of tumour biology including cancer-induced angiogenesis ( Britto *et al*., 2018; Haldi *et al.*, 2006; Nicoli *et al*., 2007); cancer cell invasion and metastasis ( de Boeck *et al.*, 2016; Marques *et al.*, 2009); cancer stem cell growth ( Bansal *et al.*, 2014; Chen *et al.*, 2017); interaction of cancer cells with the host ( Feng & Martin, 2015); and drug screening ( Corkery *et al.*, 2011; Gibert *et al.*, 2013). Importantly, the development of human tumours and their response to chemotherapeutic treatment in zebrafish embryos is comparable to that observed in mouse xenograft assays ( Fior *et al.*, 2017). Additionally, while mouse xenograft models require immuno-deficient mice to prevent immune-rejection of the human cancer cells, the lack of a mature adaptive immune system within zebrafish embryos up to 14 dpf allows analysis of human cancers without rejection ( Lam *et al.*, 2004).

In this article we describe the techniques for performing embryonic zebrafish xenograft experiments and demonstrate the utility of using zebrafish embryos as a model system for studying human cancer metastasis, in particular metastatic melanoma and prostate cancer. We highlight the advantages over mouse xenograft models and provide a practical experimental protocol showing how zebrafish embryos can be used as a replacement for mice to conveniently study metastatic tumour behaviour in the laboratory.

## Methods

A full step-by-step protocol can be found in Supplementary File 1.

### Zebrafish husbandry

Transparent *Casper* Tg( *kdrl;GFP)* zebrafish were housed under standard conditions at 28.5°C ( Westernfield, 2000). All animals were maintained under UK Home Office project licence 604548 according to the requirements of the Animals (Scientific Procedures) Act 1986 of the UK Government and conformed to Directive 2010/63/EU of the European Parliament. Zebrafish eggs were collected by timed pair mating and incubated in E3 media at 28.5°C in air until 48 hours post fertilisation (hpf). A completed ARRIVE checklist can be found in Supplementary File 2. Embryos are maintained under anaesthesia where appropriate and killed prior to 120 hpf using a schedule 1 method. For individual embryos this can be through destruction of the brain using forceps, or for larger numbers destruction of the brain can be assured using a polythene rolling pin.

### Human cell culture

Human melanoma cells A375 (American Type Culture Collection (ATCC), Manassas, USA; RRID, CVCL_0132), as well as C8161 (RRID, CVCL_6813) and WM164 (RRID, CVCL_7928) (generously gifted by Professor Meenhard Herlyn, The Wistar Institute, Philadelphia, USA), or PC-3M-Pro4-mCherry prostate cancer cells (ATCC; RRID, CVCL_D579), were incubated at 33°C for 24 hours to precondition cells prior to staining with 1,1′-Dioctadecyl-3,3,3′,3′-tetramethylindocarbocyanine perchlorate (DiI; Vybrant red fluorescent dye; Invitrogen, Paisley, UK) and injection into zebrafish embryos.

### Injection of cancer cells into zebrafish embryos

Zebrafish embryos at 2 dpf were immobilised using 1.2 mM tricaine methanesulfonate, which is a water soluble, fast-acting anaesthetic agent. Zebrafish embryos were then embedded in a thin film of low-melting-point agarose to stabilise the fish in a lateral position. To investigate invasion of cancer cells from the extravascular compartment into the vasculature, approximately 250 Dil-labelled melanoma cells in a volume of 5 nl were injected into the inferior section of the yolk sac. Similarly, to investigate tissue tropism of cancer cells, 250 DiI-labelled prostate cancer cells in a volume of 5 nl were injected into the vein of Cuvier. Following injection, fish were carefully removed from the agarose/tricaine solution using Dumont No5 fine forceps and transferred individually into 96-well plate imaging chambers created from 1% agarose using 3D printed pins ( Wittbrodt *et al.*, 2014). Minor orientation was required and embryos were suitable for microscopic analysis within 2 hours of injection.

### Confocal microscopy

Confocal images (250 μm total z-depth) of each fish were captured at 0, 24 and 72 hour time points, or every 15 mins for 5 hours for time-lapse imaging, using an inverted Leica SP8 confocal microscope (Leica Microsystems, GmbH Heidelberg, Germany) at 405 nm (blue FluoSpheres), 488 nm (green blood vessels) and 564 nm (red cells). The movement of DiI-positive melanoma cells was analysed using Volocity 3D Image Analysis Software (Volocity 6.3; PerkinElmer, Waltham, Massachusetts, USA) by manually measuring the two-dimensional distance moved by individual melanoma cells from site of injection. This analysis could alternatively be performed using ImageJ to measure the calibrated pixel distance. The number of RFP-expressing prostate cancer cells was analysed using ImageJ software version 1.8.0_112 ( https://imagej.nih.gov/ij) to quantify the total area and intensity of RFP fluorescence.

### Statistical analysis

For the analysis of tissue-specific homing of prostate cancer cells, 2 dpf zebrafish embryos from a pool of embryos derived from several mated adult zebrafish pairs were randomly assigned to receive an injection of PBS, or an injection of cancer cells, into the vein of Cuvier. The experimental unit is the individual zebrafish embryo, and a sample size of 4 embryos per group was selected on the basis of a normal standard deviation set at 95% confidence level (z = 1.96), a confidence interval (c) of 0.05 and assuming an effect size of 90% (p = 0.9) based on pilot experiments, according to the formula: n = (2z(p)(1-p))/2c. Measurement of total RFP-fluorescence within confocal images was performed and analysed using two-tailed Student’s t-test by a second researcher using GraphPad Prism 7 software (Graph Pad, San Diego, CA USA).

## Results

### Local non-vascular metastatic spread

**Figure 1.**
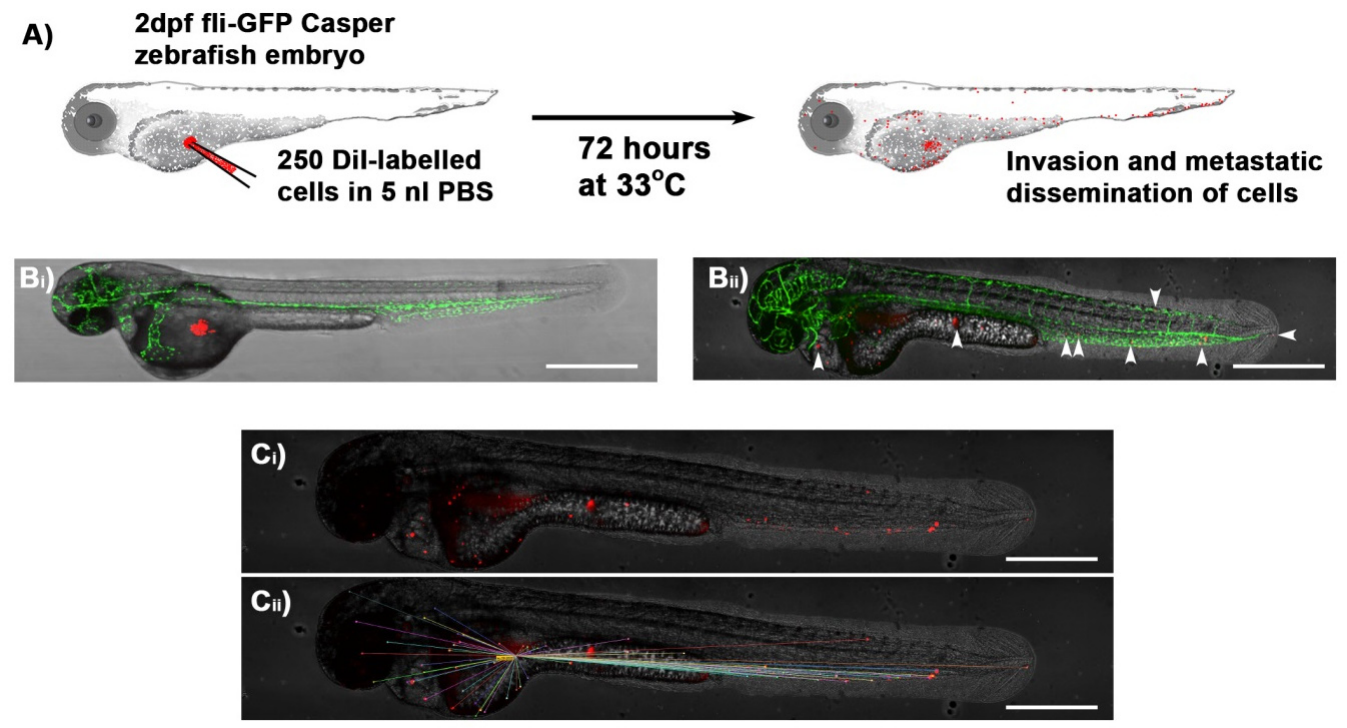
Schematic of xenograft assay and analysis of cell migration. **A**) Site-specific injection (depicted into the yolk sac) of DiI- or RFP-labelled (Red) cancer cells in 5 nl PBS into 2 dpf zebrafish embryos is followed by incubation of zebrafish for 72 hours at 33°C and subsequent imaging analysis of invasion and metastatic dissemination of cancer cells. **B**) Approximately 250 DiI-labelled A375 melanoma cells 0 hrs ( **Bi**) and 72 hrs ( **Bii**; white arrows indicate position of melanoma cells) after injection into the yolk sac of Tg(kdrl-GFP) *Casper* zebrafish (Green blood vessels). **C**) Confocal z-stack images are used to visualise red DiI fluorescence of melanoma cells within zebrafish ( **Ci**) and the distance from injection site measured using Volocity image analysis software ( **Cii**); Scale bar = 500 μm.

The initial event in metastatic spread is the movement of an individual cancer cell from the tumour niche. This can be modelled using *in-vitro* systems such as skin organoids or the Dunn chemotactic chamber, but neither of these assays are suitable for measuring metastasis. In our zebrafish embryo xenograft model, we inject small deposits of fluorescently labelled human cancer cells into the yolk sac at 2 dpf, and track individual cells until 5 dpf ( Figure 1A). By using a zebrafish line with absent pigmentation it is possible to achieve excellent views throughout transgenic embryos with GFP-labelled endothelial blood vessels (green; 510 nm emission), ensuring injection of DiI-labelled A375 melanoma cells (red; 565 nm emission) into the extravascular compartment ( Figure 1Bi), which directly migrate to peripheral sites ( Figure 1Bii. Although embryos are normally allowed to develop at 28.5°C and human cells at 37°C, a compromise at 33°C works well. The movement of individual melanoma cells from site of injection can be measured using ImageJ or Volocity image analysis software ( Figure 1C).

### Intravasation of metastatic cells

**Figure 2.**
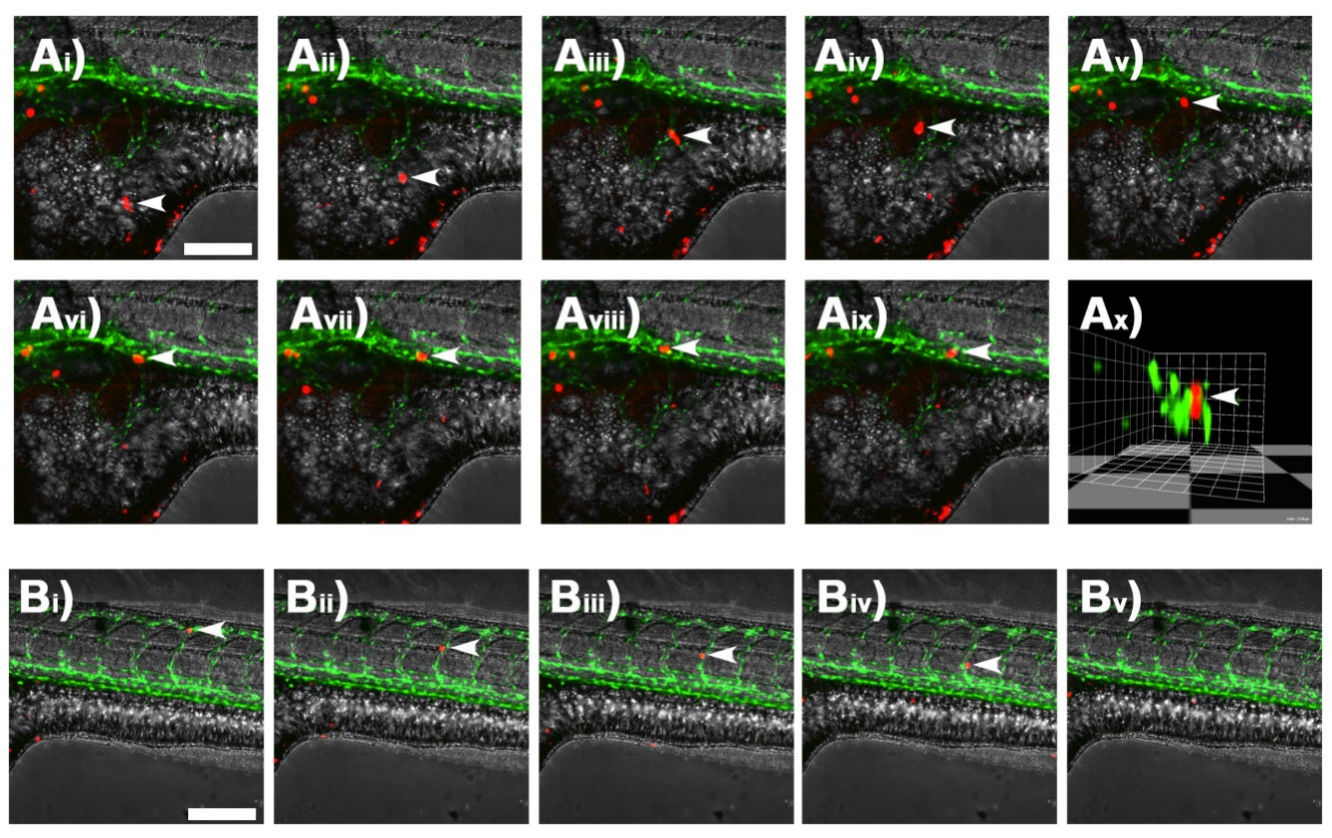
Single cell tracking by time-lapse confocal microscopy. **Ai**– **ix**) Confocal z-stack images taken at 15 minute intervals showing an individual DiI-labelled A375 melanoma cell (white arrows) migrating within the yolk sac of a casper zebrafish embryo and interacting with a GFP-tagged blood vessel. **Ax**) 3D-render of image *Aix* rotated to show the transverse section through the GFP-tagged blood vessel with DiI-labelled melanoma cell indicated by white arrows. **Bi**– **v**) Confocal z-stack images taken at 15 minute intervals showing an individual DiI-labelled melanoma cell (white arrows) within the GFP-tagged blood vessels of a casper zebrafish embryo. Scale bar = 150 μm.

The ability to carry out time-lapse imaging on embryos affords the opportunity to examine individual cell movement. Injected embryos were lightly anaesthetised using tricaine and orientated in low-melting-point agarose. By focusing on the point of injection, DiI-labelled melanoma cells were visualised moving through the extravesicular compartment within the yolk sac of zebrafish embryos using low-voltage time-lapse confocal microscopy ( Figure 2A and Supplementary Movie 1). A 3D-rendering of the confocal image z-stack was rotated to reveal the transverse section of the blood vessel showing a melanoma cell positioned between the zebrafish endothelial cells, indicating that this cell is directly within the blood vessel ( Figure 2Ax and Supplementary Movie 2).

### Metastasis and the endothelium

Haematological or lymphatic metastatic dissemination requires interaction with the endothelium during entry and exit. However, patients can also have cancer cells circulating in their blood that do not necessarily show metastases ( Reymond *et al.*, 2013). It is now recognised that metastasising cells exhibit sticking and rolling as they interact with the endothelium, and surface molecules such as selectins and CD44 are implicated. The zebrafish embryo xenograft model shows potential to be an extremely powerful tool in understanding the relationship between the surface biology of tumour and endothelial cells. Time-lapse confocal microscopy at 15-minute intervals readily captured melanoma cells as they demonstrated sticking and rolling behaviours on the surface of vascular endothelium ( Figure 2B and Supplementary Movie 3) clearly suggesting a specific interaction of the human melanoma cells with zebrafish endothelial cells.

**Figure 3.**
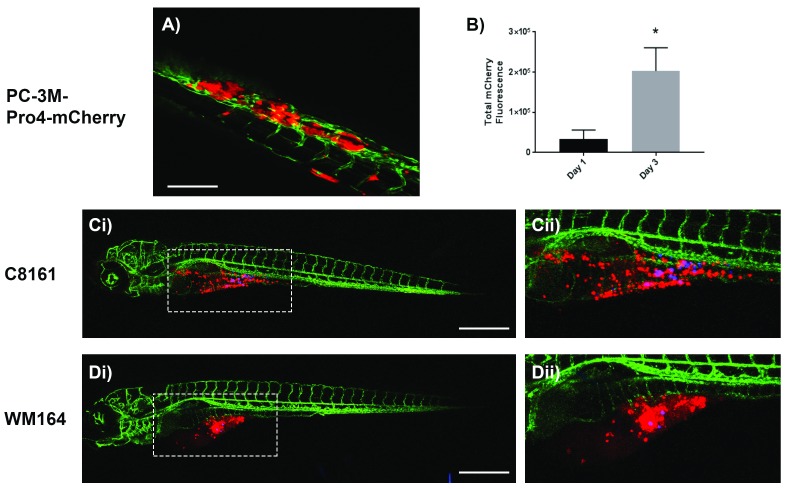
Representative confocal z-stack images of kdrl-GFP casper zebrafish embryos 72 hours after injection with human cancer cells. **A**) PC-3M-Pro4-mCherry prostate cancer cells injected into the duct of Cuvier form tumours in the caudal hematopoietic tissue of the zebrafish tail; Scale bar = 150 μm. **B**) Quantification of total mCherry fluorescence by prostate cancer cells after 1 and 3 days post injection; n=4, *p<0.01, 0.05 CI, paired t-test. **Ci**– **ii**) C8161 and **Di**– **ii**) WM164 melanoma cells (stained with Red DiI dye) injected alongside FluoSpheres (Blue) into the yolk sac survive and invade throughout the yolk sac; Scale bar = 500 μm.

Tumour cells can also be directly injected into the circulation of developing zebrafish via the vein of Cuvier providing a tractable model of metastatic cancer cell-endothelial interaction during vascular exit. This is particularly important in some tumours that do not readily enter the vasculature, but do have tissue tropic exit routes. For example, the prostate cancer cell line PC-3M-Pro4-mCherry does not metastasise from the yolk sac, but when injected into the circulation these cells seed in the caudal hematopoietic tissue of the zebrafish tail where they proliferate, suggesting a specific microenvironmental niche favourable for tumour development ( Figure 3A, B).

### Heterogeneity of metastatic cell behaviour

A specific benefit of embryonic zebrafish over other larger preclinical laboratory models is that several experiments can be carried out in parallel on the same microscope stage. This allows screening of a library of pharmacological candidates, but importantly evaluation of different metastatic cell types, which may be primary cell lines derived directly from patients. This is important as heterogeneity between or within patient tumours may be important in metastatic behaviour. We have seen this in our own melanoma work, where C8161 cells disseminated widely throughout the yolk sac ( Figure 3C) while WM164 cells formed a localised tumour-like mass with fewer melanoma cells invading the yolk sac ( Figure 3D). Co-injection of tracer beads can be used to distinguish passive developmental associated movement from active migration and invasion of cancer cells. Metastatic A375 cells were found in the distal tail vessels, whilst very few C8161 and WM164 cells were found in the tail and other regions of the zebrafish by 72hpf, indicating C8161 and WM164 cells have a reduced capacity to invade blood vessels, which may limit their metastatic potential.

These vital imaging-based assays used in combination with the ability to genetically modify zebrafish or apply pharmacological agents represent important new tools and approaches to understand these metastatic processes at a cellular level.

Raw images used to generate figures shown in this study Shown are images for Figures 1 and 3; images in Figure 2 were obtained from stills of Supplementary Movies 1-3. Click here for additional data file. Copyright: © 2018 Hill D et al. 2018 Data associated with the article are available under the terms of the Creative Commons Zero "No rights reserved" data waiver (CC0 1.0 Public domain dedication).

## Discussion

We and others have shown that xenotransplantation of human cancer into zebrafish embryos can be optimally carried out at 48 hfp when gastrulation is complete and the main body plan of the animal is established. The next 72 hours provides sufficient time frame to model key stages of metastatic behaviour, including local invasion, vascular entry, circulation and vascular exit. In this paper we have demonstrated how tumour invasion and/or metastatic dissemination by human cancer cells can be monitored through time-lapse microscopy. Most importantly, metastatic processes of single cells can be visualised at the earliest time points, which is not possible in a mouse model. The zebrafish embryonic xenograft of human cancer therefore directly replaces the need for using mouse xenografts and avoids welfare concerns associated with mouse models, including pain and suffering due to unexpected or excessive primary tumour growth. In the UK alone, it is estimated that over 550,000 mice are used each year for cancer research (UK Home Office statistics). On average 50 mice are used per study of cancer metastasis, and over the past 5 years there have been on average 900 publications per year in this area. We therefore estimate that 45,000 mice are used each year for research of cancer metastasis using mouse xenograft models, many of which could be replaced by embryonic zebrafish at unregulated stages of development, using the model described in this paper. To do this several historical concerns need to be addressed.

Experimentally, it is essential that following xenotransplantation human cells can be distinguished from host cells of the zebrafish. Whilst this can be achieved *post-mortem* by detecting human-specific antigens using immunocytochemistry ( Bentley *et al.*, 2015), the use of lipophilic fluorescent cell membrane stains in conjunction with zebrafish transgenic lines allows visualisation of cells both during time-lapse imaging and after tissue fixation. These methods provide equivalent quantification of xenografted cancer cell proliferation ( Bentley *et al.*, 2015).

It might be suggested that differences in cell size, microenvironmental niches and molecular signalling pathways between human patients and preclinical models (mice as well as zebrafish) could limit the relevance and translational value of data obtained from animal studies. However, our studies show that human cancer cells are able to invade zebrafish blood vessels and form secondary tumours, which can be inhibited by specific autophagy inhibition ( Verykiou *et al*., 2018); while previous studies have shown that VEGF and CXCR4 signalling are conserved between human cancers and zebrafish ( He *et al.*, 2012; Tulotta *et al.*, 2016). Nevertheless, further studies to characterise the response of human cells in the zebrafish model organism are required.

The future direction of research using zebrafish embryos for human xenograft studies will likely focus on strategies and methods to increase assay throughput and improve analysis of large data sets. These objectives will benefit from a number of technical innovations, such as devices to orientate the zebrafish for imaging ( Wittbrodt *et al.*, 2014) as well as automated quantification and analysis of tumour cell dissemination ( Ghotra *et al.*, 2012; Heilmann *et al.*, 2015). The analysis of in-situ cancer cell proliferation could be improved by using techniques such as EdU incorporation or use of a transgenic cell cycle reporter such as FUCCI within cancer cells ( Haass *et al*., 2014)

Stable cancer cell lines are often dramatically different from patient tumour cells and by definition have been selected for ease of maintenance in the laboratory environment. However, it is likely that heterogeneity and cooperation of cancer cells in patient tumours drives tumour invasion through remodelling of the extracellular matrix ( Chapman *et al.*, 2014). Thus, cancer is represented by cells that vary in their proliferative, invasive and metastatic phenotype, which contributes both to tumour growth and also emergence of drug resistance ( Anderson *et al.*, 2011). However, it is often not feasible to investigate the effect of tumour cell heterogeneity in mouse xenograft models as large numbers of patient primary tumour cells are required for successful engraftment. In contrast, a major advantage of the embryonic zebrafish xenograft assay is the capacity to accurately detect and monitor a small number of cells (100–200 cells per fish), including low-number cancer subpopulations such as cancer stem cells, drug-resistant cells or primary patient tumour tissue where only small numbers of cells can be recovered e.g. circulating tumour cells. Patient-derived tumour xenograft models are therefore a potential solution to the problem of limited intratumoural heterogeneity of cell line derived xenografts, which may also improve the accuracy of tumour drug-response studies.

It is becoming increasingly clear that no single pre-clinical model can substitute for actual human trials, and therefore as researchers we must continually reassess and adapt our model assays to improve their relevance, which will likely involve employing an approach that combines multicellular *in vitro* organoid assays ( Hill *et al.*, 2015) with both zebrafish and mouse *in vivo* studies. We suggest that this combinatorial approach will reduce the reliance on mouse xenograft models for the study of human cancer metastasis and drug screening. However, the challenge for translational cancer research will be to integrate the multitude of data from different model organisms to identify evolutionary conserved drug-tumour interactions between species so that we may select the most appropriate therapeutics that have the highest chance of providing an effective treatment for patients with cancer.

## Data availability

The data referenced by this article are under copyright with the following copyright statement: Copyright: ï¿½ 2018 Hill D et al.

Data associated with the article are available under the terms of the Creative Commons Zero "No rights reserved" data waiver (CC0 1.0 Public domain dedication). 

**Dataset 1. Raw images used to generate figures shown in this study.** Shown are images for Figure 1 and Figure 3; images in Figure 2 were obtained from stills of Supplementary Movie 1– Supplementary Movie 3. DOI: https://doi.org/10.5256/f1000research.16659.d221978 ( Hill *et al*., 2018).

## Supplementary material

**Supplementary File 1. Complete step-by-step protocol for Zebrafish xenograft of human cancer cells.**

**Supplementary File 2. Completed ARRIVE checklist.**

**Supplementary Movie 1. Representative time lapse confocal movie showing active invasion of a DiI-labelled A375 melanoma cell through the yolk sac and into a kdrl-GFP labelled blood vessel of a Casper zebrafish embryo.**

Images were taken every 15 minutes for 5 hours.

**Supplementary Movie 2. 3D rendering of confocal movie showing active invasion of a DiI-labelled A375 melanoma cell through the yolk sac and into a kdrl-GFP labelled blood vessel of a Casper zebrafish embryo.**

Transverse field of view through the blood vessel demonstrates that the red melanoma is within the green blood vessel and actively moving against the flow of blood.

**Supplementary Movie 3. Representative time lapse confocal movie showing movement of a DiI-labelled A375 melanoma cell through a kdrl-GFP labelled blood vessel of a Casper zebrafish embryo.**

Images were taken every 15 minutes for 5 hours.
