# Supplementary material for: Embryonic zebrafish xenograft assay of human cancer metastasis
Source: F1000Res. 2018 Dec 20;7:1682. Originally published 2018 Oct 22. [Version 2] doi: 10.12688/f1000research.16659.2 (PMC6234738; doi:10.12688/f1000research.16659.2)
Supplement: Supplementary file 2 [file f1000research-7-18962-s0001.tgz › 1eae9a10-b2e3-4762-b033-515d084709c9_Supplementary_File_1_Step_by_step_protocol_v2.docx]

**Protocol for Zebrafish Xenograft of Human Cancer Cells**

**Materials required**

**Breeding zebrafish and collecting eggs**

Petri dishes, 15 ml tubes and plastic pipettes (Thermofisher Scientific, Paisley, UK)

E3 media (60X stock: 172g NaCl, 7.6g KCl, 29g CaCl_2_.2H_2_O, 49g MgSO_4_.7H_2_O in 1L ddH_2_O) and methylene blue (Sigma-Aldrich Ltd, Poole, UK)

28.5^o^C incubator (e.g. Heraeus B15; Thermofisher Scientific, Paisley, UK)

**Culturing and staining human cancer cells (A375, C8161, WM164 melanoma cells; PC-3M-Pro4 prostate cancer cells)**

T75 tissue culture flask (Thermofisher Scientific, Paisley, UK)

High-glucose Dulbecco’s Modified Eagles Medium with L-glutamine (DMEM; Lonza, Vervies, Belgium)

Foetal calf serum (FCS; Sigma-Aldrich Ltd, Poole, UK)

Penicillin/streptomycin (P/S; Lonza, Vervies, Belgium)

37^o^C incubator and 33^o^C incubator

DiI Vybrant red fluorescent dye (V22885; Invitrogen, Paisley, UK)

PBS and Trypsin/EDTA (ThermoFisher Scientific, Paisley, UK)

FluoSpheres (365/415 nm, 15 μm size, F8837; Invitrogen, Paisley, UK)

**Injection of cancer cells into zebrafish embryos**

Nanoliter 2000 injector system (092512; World Precision Instruments, Hertfordshire, UK)

Tricaine (15 mM stock: 400 mg ethyl 3-aminobenzoate methanesulfonate, 0.26 g Tris, 100 ml 1X E3 media. Adjust to pH 7; Sigma-Aldrich Ltd, Poole, UK).

Low-melting point agarose (R0801; Thermofisher Scientific, Paisley, UK)

Borosilicate glass capillaries (1B200-6; World Precision Instruments, Hertfordshire, UK)

Micropipette puller (Model P-97; Sutter instruments Co)

Ultrafine forceps (EROP3CSA-ND; Apex Tool Group; Digi-Key electronics)

Fluorescent stereo Leica M165 FC microscope (Leica Microsystems, GmbH Heidelberg, Germany)

**Imaging of injected zebrafish embryos**

3D printed mould to create imaging chambers (Wittbrodt *et al* 2014; doi.org/10.1186/1472-6750-14-36)

Low-melting point agarose (R0801; Thermofisher Scientific, Paisley, UK)

96-well plate (Thermofisher Scientific, Paisley, UK)

Inverted Leica SP8 confocal microscope (Leica Microsystems, GmbH Heidelberg, Germany)

**Planning**

**Zebrafish**

Many studies can be carried out using a wildtype zebrafish line, such as AB or Tu strains. As the zebrafish embryos are used prior to protected stages, no regulatory documentation is required, but it is good practice to discuss your experiment with the Named Animal Care Welfare Officer (NACWO). It is usually possible to use surplus eggs from other zebrafish researchers, obtained as part of normal husbandry. This directly reduces the number of animals used in research and removes the expense of keeping animals. It is important to ensure that experiments end before regulated stages (in the UK zebrafish are protected by the Animal (Scientific Procedures) Act after 5 days post fertilisation); you may need to apply for a project licence to maintain zebrafish lines that are specific to your experiments.

**Experiments**

Experimental planning can ensure that the minimum number of animals are used in experiments, but also that you do not waste time and reagents. We recommend writing out experiments including statistical analysis. If there is no data for power calculations, pilot studies should be carried out. The NC3Rs Experimental Design Assistant (https://www.nc3rs.org.uk/experimental-design-assistant-eda) is a useful tool for creating a study plan and also for determining each experimental group sample size. An example study plan outlining a potential drug treatment experiment can be seen in Figure A. A review of the experimental design by an experienced colleague can be invaluable in improving experimental design and reducing numbers of animals used.

**Reagents**

Before injecting cells into embryos, it is sensible to check pharmacological agents for toxicity, e.g Tricaine. Use a range of concentrations and choose agents that have been proven to cross the skin of zebrafish embryos.

**Injection needles**

Microinjection needles can be made from borosilicate glass capillaries (1mm without filament) using micropipette puller (Model P-97; Sutter instruments Co) set to maintain a large luminal cross-section. Typical settings are P=200, Heat=638, Pull=100, Velocity=80, Delay=200.

**Human cells**

Ensure the human cells are healthy and growing well. You should know the replication rate is as expected and ensure they are not infected. When you isolate the cells for injection, keep a portion in the cell culture facility to ensure they are healthy. Confirm identity of cell lines with short tandem repeat (STR) profiling and regularly test for mycoplasma contamination.

**Injection moulds**

Although injection moulds can be produced with a desktop heat-extrusion 3D printer, we found that the smoother surface produced, for example by selective laser sintering, from a commercial printing company, allowed better release from the agarose. Typically, their design departments can help with implementing published designs ([Wittbrodt, Liebel et al. 2014](#_ENREF_1)) and a smaller e.g. 24 pin pattern can be produced if fewer animals are needed.

**Experimental procedure**

**Breed zebrafish and collect eggs.**

TIMING: Day 0, 30 mins – 1 hour.

1. Research facilities will use different methods to breed zebrafish. After eggs are laid, return adult fish to tanks and inspect eggs to ensure only high-quality clutches off eggs are to be used in xenograft experiments. A healthy pair of adult zebrafish will usually lay 100 – 200 eggs per a mating, and we expect over 90% of eggs to be fertilised and develop normally over the next two days. Although the unit of experiment will be the individual embryo, we make sure that embryos from several different clutches are used. In addition, 20 – 50 embryos are maintained separately in a petri dish containing 1X E3 media to ensure healthy development of each pool of zebrafish at optimal temperature of 28.5^o^C and normal atmosphere for the duration of the experiment.
2. Gently wash eggs using system water (optionally containing methylene blue) and transfer approximately 50 eggs to a petri dish containing 1X E3 media. TROUBLESHOOTING i.
3. Transfer eggs to an incubator at 28.5^o^C for 48 hours.

**Remove dead or infected eggs.**

TIMING: Day 1, 30 mins – 1 hour.

1. Use a stereo microscope to identify and remove dead or infected eggs, which turn opaque following coagulation of the embryo or infection with fungi or vorticella.

**Culture human cancer cells.**

TIMING: Day 1, 30 mins – 1 hour.

1. Seed 2x10^6^ human cancer cells per T75 tissue culture flask in 10 ml high glucose (4.5 g/L) Dulbecco’s Modified Eagles Medium with L-glutamine (DMEM) supplemented with 10% foetal calf serum (FCS) and 5% penicillin/streptomycin (P/S) (complete culture medium).
2. Incubate cells at 33^o^C in a humidified atmosphere of air with 5% CO_2_ for 24 hours.

**Stain human cancer cells.**

TIMING: Day 2, 1 hour.

1. Replace media on human cancer cells with 5ml complete culture media containing 25 μl DiI and incubate at 33^o^C for 20 minutes. Aspirate media containing dye and wash 3 x 10 mins with warm culture media at 33^o^C. Leave cells in final wash media until use. TROUBLESHOOTING ii.

OPTIONAL: We recommend that tracer beads be used to label the original injection site and to distinguish active tumour cell migration from passive movement that occurs when tissues and organs within the yolk sac grow. If injected into the yolk sac, these should not be found in distal sites or within the vasculature. Place 50 μl (approx. 2 x 10^5^) of 365/415 FluoSpheres (15 μm size) into a 15 ml tube and wash in 10 ml PBS. Centrifuge at 1200 RPM for 5 mins, aspirate PBS and add 1 ml complete culture media.

**Make agarose-imaging chambers.**

TIMING: Day 2, 30 mins.

1. Add 125 mg low-melting point agarose to 25 ml 1X E3 media and microwave on full power for 2 min, or until dissolved to create a 1% agarose solution. Place melted agarose and tricaine stock in water bath at 35^o^C. When both are at 35^o^C, add 2 ml tricaine stock solution to agarose.
2. Create imaging chambers by adding 100 μl of 1% agarose/tricaine into each well of a 96 well plate and insert 3D printed pins to create an agarose mould for rapid orientation and screening of zebrafish embryos.

**Anaesthetise and immobilise zebrafish embryos in agarose.**

TIMING: Day 2, 30 mins.

1. If zebrafish embryos have not hatched by 48 hours post fertilisation, remove the chorion manually from about 25-50 embryos by pulling on the chorion with forceps to release the fish.
2. Transfer approximately 50 zebrafish embryos using a 1.5 ml plastic pipette into a petri dish containing 50 ml 1X E3 media. Add 4ml tricaine stock to anaesthetise zebrafish embryos.
3. Transfer up to 25 anaesthetised zebrafish to agarose/tricaine solution at 35^o^C in the lid of a petri dish. Quickly orientate fish so it is lying on its side with head pointing right, and then remove excess agarose so that fish become embedded in a very thin film of agarose (Figure B). When set, put 1X E3 media containing 1.2 mM tricaine onto the agarose.

**Harvest human cancer cells.**

TIMING: Day 2, 30 mins.

1. When zebrafish embryos are anaesthetised and embedded in agar, cancer cells can be harvested. Wash DiI-stained or fluorescently-labelled cancer cells briefly with PBS, and aspirate. Add 1.5 ml Trypsin/EDTA and put flask at 33^o^C until cells are released.
2. Re-suspend cells in 10 mls culture media. Count cells using a slide haemocytometer, and transfer 2 x 10^6^ cells to a 15 ml tube (with fluorosheres if required at 1:10 ratio). TROUBLESHOOTING iii.
3. Centrifuge at 1200 RPM for 5 minutes, tip off supernatant, re-suspend in minimal residual media and adjust volume to 40 μl with fresh culture media. TROUBLESHOOTING iv.

**Set up microinjector.**

TIMING: Day 2, 30 mins.

1. Set up manual microinjector (e.g. Nanoliter 2000 injector system) to dispense a volume of 5 nl and attach needle at approximately 45^o^ angle. Break needle to a diameter of 30-40 μm while attached to the microinjector using a pair of ultrafine forceps. The needle should be sharp with no jagged edges so that it pierces the skin of the zebrafish with minimal tissue damage, which would result in efflux of the cells following injection (Figure C). TROUBLESHOOTING v.
2. Transfer 20 μl of cell/fluorosphere suspension to a depression slide. Aspirate cell and fluorosphere suspension into microinjection needle using the vacuum function of the microinjector (Figure D).

**Inject cancer cells into zebrafish embryos.**

TIMING: Day 2, 30 mins – 1 hour.

1. Use a stereo microscope to position zebrafish so that the injection site is directly under the needle. Move the needle forward using the manipulator tools on the microinjector to pierce the skin of the zebrafish and penetrate until the tip of the needle is in the desired location. For example, to inject into the yolk sac insert needle into the rear half of the yolk sac to a depth of 50% of the width of the yolk sac (See Figure 1A of manuscript). Cells can also be injected into other target sites such as brain, heart, vein of Cuvier etc.
2. Depress the foot-pedal to inject 5 nl of suspension and keep needle in place briefly before removing to ensure cell suspension is not forced back out. TROUBLESHOOTING vi.
3. Inject twice as many embryos as will be needed and choose optimally injected embryos. Carefully remove anaesthetised fish embryos from agarose by disrupting the agarose surrounding the fish using forceps without touching the fish itself. The fish will be easily released into the media without much force when the agarose is broken up.Check for the presence of cells using a fluorescent stereo microscope through a dsRed filter. Discard any fish with cells outside the injection site, at multiple sites or without cells. Initially only a few embryos may be acceptable, but within 2-3 sessions 30-40 zebrafish embryos can be successfully injected. TROUBLESHOOTING vii.

**Take images of injected zebrafish embryos at 0 hour time point.**

TIMING: Day 2, 2 hours.

1. Using the Randomised Sequence Allocation tool within the NC3Rs Experimental Design Assistant, assign the required number of fish at random to each treatment group (If the subsequent experimental analysis is to be performed by another researcher the randomisation sequence can be emailed to them so that the analysis can be blinded).
2. Transfer zebrafish embryos individually using a 1.5 ml plastic pipette to imaging chambers created during Step 9 in 150 μl 1X E3 media containing 1.2 mM tricaine and carefully orientate each fish into position with all fish aligned in the same direction (Figure E).
3. Using a confocal microscope capture full-depth images of each zebrafish embryo using a separate acquisition channel for cancer cells, fluospheres (if included), and any other fluorescent reporters present in the zebrafish. *High-resolution images are not required to monitor metastasis but single cells must be distinguishable.* Make a note of the microscope settings used, including magnification, z-step size, laser power, gain and off-set etc.
4. Place imaging chambers containing anaesthetised zebrafish embryos into an incubator at 33^o^C for 72 hours.

OPTIONAL: We have never observed any detrimental effects, such as developmental abnormalities, to the zebrafish embryos after 72 hrs treatment with 1.2 mM tricaine. However, the zebrafish embryos can be revived by carefully removing tricaine-containing E3 media using a pipette and adding 150 μl 1X E3 media only.

**Take images of injected zebrafish embryos at 72 hour time point and terminate experiment.**

TIMING: Day 5, 2 hours.

1. Using the same confocal microscope setting used during Step 20, capture full-depth images of each zebrafish embryo using a separate acquisition channel for cancer cells, fluorospheres (if included), and any other fluorescent reporters present in the zebrafish. TROUBLESHOOTING viii.
2. Prior to 120 hpf, terminally anaethetise zebrafish embryos using tricaine and ice cold fixation, e.g. using 4% paraformaldehyde, for further analysis. Or alternatively, destroy the brain using forceps or a polythene rolling-pin.

**Troubleshooting**

1. Step 2: If assay contains dyes or labels with blue or UV fluorescence then omit methylene blue as this will increase the background fluorescence at these wavelengths. E3 media or tank water can be used instead to wash eggs.
2. Step 7: Labelling efficiency is usually 100%, but don’t leave the cells in DiI for longer than 20 minutes as this will result in excessive staining that will leach out into the surrounding zebrafish tissue following injection, which will increase background fluorescence.
3. Step 14: Use only warm culture media to wash DiI stain from cells as heat shock at this point can affect invasive and metastatic behaviour of the cells.
4. Step 15: It is also important that cancer cells are *not* left on ice prior to injection as this can also affect the invasion and metastasis of cells. It is advisable to anaesthetise and embed the zebrafish embryos (Steps 10-12) immediately after staining cancer cells but prior to harvesting the cancer cells.
5. Step 16: Performing injections in the lid of a petri dish allows the needle to be orientated at the appropriate angle for easy injection. If fish are embedded in a dish with high sides the injections become much more difficult.
6. Step 19: Although agarose moulds can be used to avoid braking needles on the petridish, in practice it is easier to mount a new needle that to create the agarose moulds. Keep the needle horizontal when possible to prevent cells settling to the needle tip through gravity.
7. Step 20: If cancer cells settle to the bottom of the needle, rotate needle 180^o^ to redistribute cell suspension. Cancer cells *must* also be in a single cells suspension otherwise needle will block. If injecting multiple cancer cell lines, complete injections for each cell line within 1 hour and temporarily return fish to 33^o^C. Harvest cancer cell lines sequentially if necessary. Each injection takes approximately 30 seconds with approximately 50-75% success rate.
8. Step 24: The injection site can be easily identified at 72 hours if inert FluoSpheres have been co-injected with cancer cells or if cancer cells form a primary tumour mass, which invading cells can be measured relative to.

**Timing**
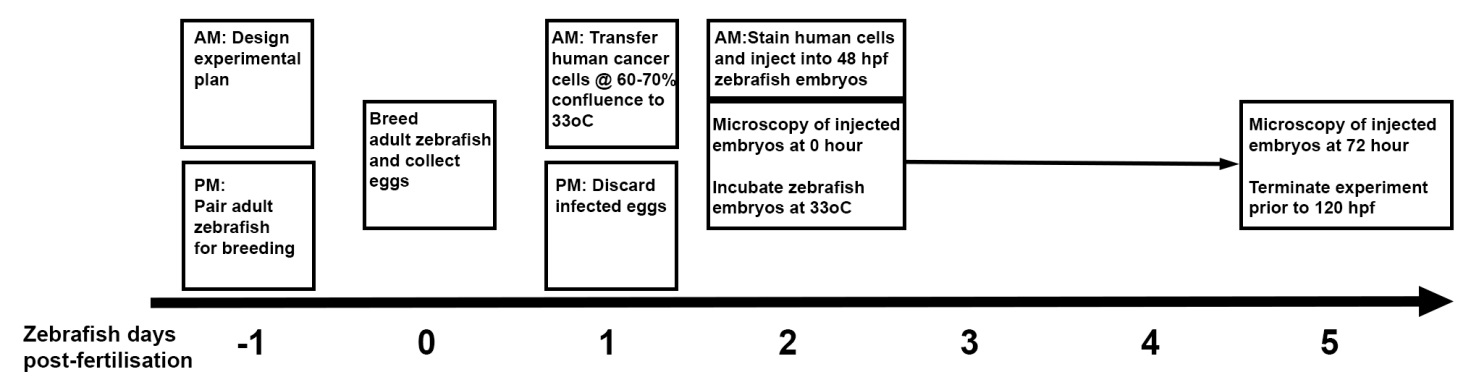


**Figures**


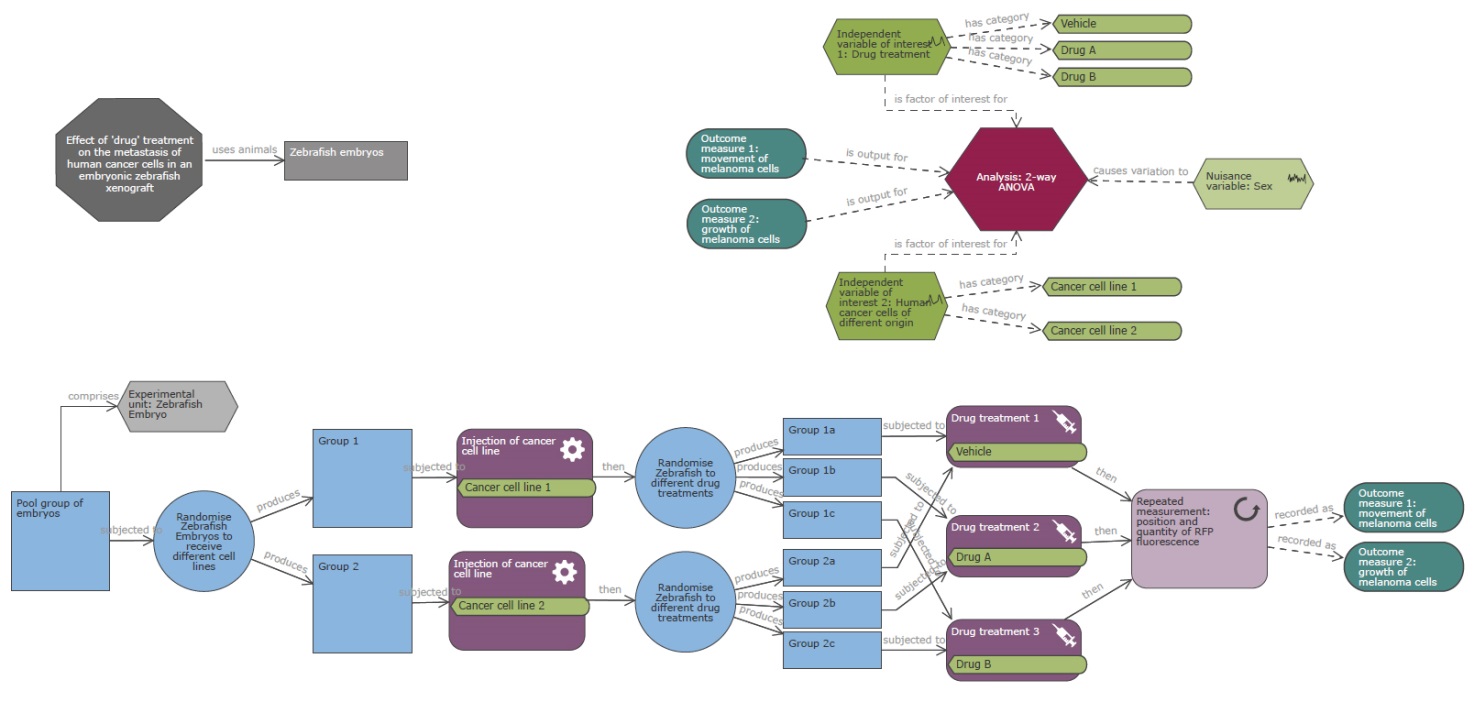


Figure A: Example of a study plan created using the Experimental Design Assistant showing a simple comparative study for the effect of two drugs on the metastatic spread of two different cancer cell lines. Block randomisation has been used to create 3 groups containing an equal number of zebrafish embryos injected with either cell line, and each group will be treated with a different drug treatment (including vehicle control). Each measurement outcome will be analysed by 2-way ANOVA to determine the effect of drug treatment on growth, survival and invasion of each cancer cell line.


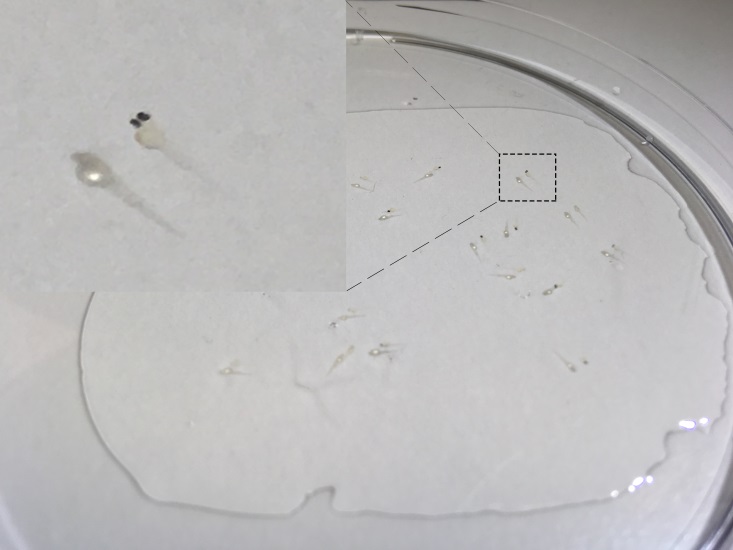


Figure B: Embed and orientate zebrafish embryos in a thin film of 1% low-melting point agarose containing 1.2 mM tricaine.


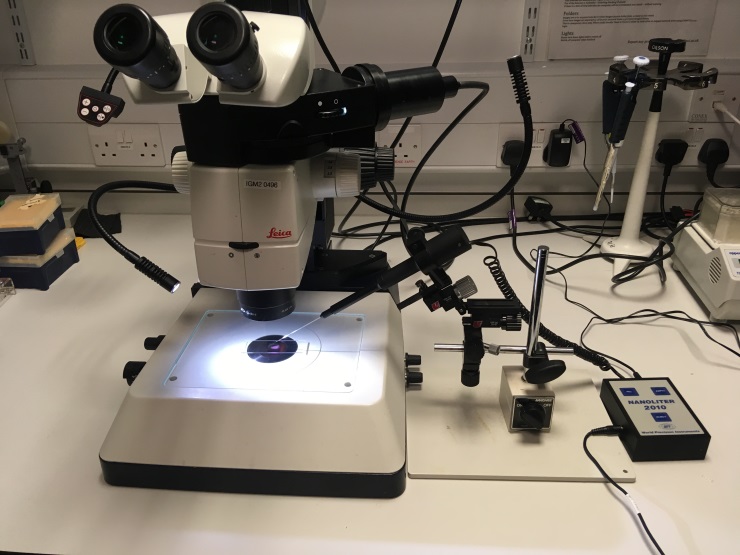


Figure C: Set up of microscope (Fluorescent stereo Leica M165 FC) and injection system (Nanoliter 2000). Use microscope to orientate zebrafish embryos in agarose, inject human cancer cells and check for the presence of cells following injection.


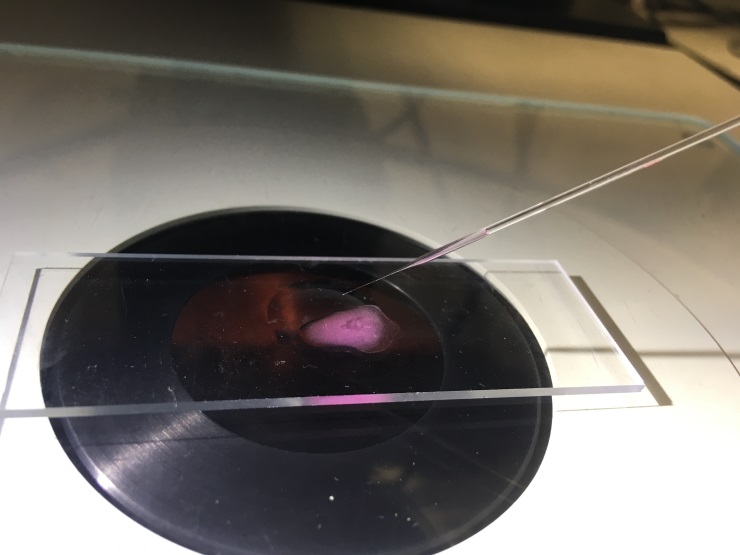


Figure D: Aspirate human cancer cells from a depression slide into the needle ready for injection into zebrafish embryos.


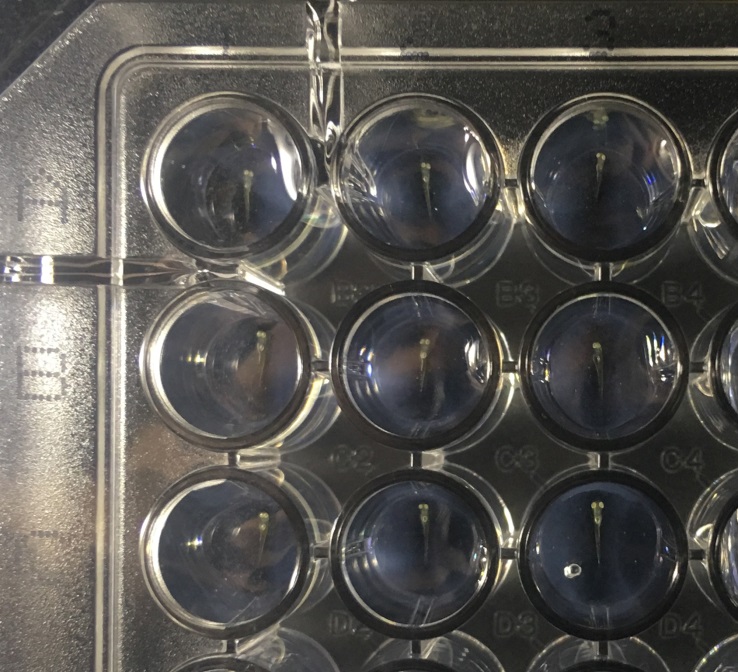


Figure E: Align and orientate zebrafish embryos in agarose imaging chambers for rapid confocal imaging.
