# Supplementary material for: Embryonic zebrafish xenograft assay of human cancer metastasis
Source: F1000Res. 2018 Dec 20;7:1682. Originally published 2018 Oct 22. [Version 2] doi: 10.12688/f1000research.16659.2 (PMC6234738; doi:10.12688/f1000research.16659.2)
Supplement: Peer review history

# Peer review: Reviewer response for version 1

**DOI:** 10.5256/f1000research.18207.r39773

**Published:** 2018-11-12

**Authors:** Yi Feng

**Affiliations:** 1 MRC/UoE Centre for Inflammation Research, Queen’s Medical Research Institute, The University of Edinburgh, Edinburgh, UK

Zebrafish embryo xenograft model is a widely adopted method to study many aspects of cancer cell behaviour in vivo. A variety of protocols that differ by injection site, incubating temperature and down stream phenotype chosen, have been used in the field. So far, there is still a lack of a publicly available, detailed standard protocol, which new users could follow. The current methodology paper provides a very detailed, easy to follow protocol for xenograft fluorescent labeled cancer cells in the yolk sac and vein of Cuvier in 2 days old embryos and how to monitor cancer cell dissemination following the graft to assess their metastasis potential. This paper would be of benefit to anyone who wants to try out the zebrafish embryo xenograft model.

 The step by step protocol could include more details that would make it more user friendly for someone who is new to fish models.

 Material section:

1. It would help if more details were given for the following reagents such as Cat number because there could be multiple products available under a similar name: Low-melting point agarose, Borosilicate glass capillaries, Ultrafine forceps. What size?, 96-well plate. Are these with a glass bottom?
2. 3D printed mould to create imaging chambers – this is referred to in another paper without any details given. Perhaps they could expand on how they made it or obtained it? The imaging chamber is a key aspect and for this protocol to be “high through-put” more details would really help the reader.

Protocol steps

1. Once embryos transferred into imaging chamber, the authors indicate that the embryos will be maintained in 150 ml 1X E3 media containing 1.2 mM tricaine for 72 hours. This could have detrimental effects on embryos. The authors should comment on whether any adverse effects were observed and how to avoid them.
2. One of the Optional steps: “Tracer beads can be used to label the original injection site and to distinguish active tumour cell migration from passive development associated movement that occurs when tissues and organs within the yolk sac grow.” To my mind it is very important for the initial set up of the model, as different cancer cell lines display different metastasis capacities and the tracer beads can be found expanding from the original injection site due to various reasons that the authors pointed out. So the use of tracer bead would help to set up a baseline index for passive expansion, true cancer cell invasion and metastasis that can then be evaluated according to the baseline parameter. The authors could perhaps comment more on their experience of using tracer beads in the main text of the paper.

The main text of the paper

 In the main text the authors nicely presented three examples of what biological features can be captured using confocal imaging analysis following graft. It would be very helpful if they could elaborate more on each of the models, presenting more details on what parameters could be established from each model.

 Model 1 (figure 1 Cii) cancer cell dissemination. It seems that 2D distance is used instead of 3D and there is no mention of the size of each cell cluster that appeared to be metastatic growth. The authors should explain more extensively why they choose such a parameter and whether there are other potential parameters that one could measure to assess dissemination of cancer cells.

 Model 2 (figure 2) intravasation and distal metastasis are extremely rare events according to other publications ( Roh-Johnson M, et al), perhaps authors could comment on how frequently they can capture intravasation or cancer cells within blood vessels? Perhaps provide some information on their experience with different cell lines in their intravasation capacity.

 Model 3 (Figure 3 A,B) injecting into the vein of Cuvier is similar to mouse tail vein injection where cancer cells are grafted directly into the blood stream. This allows for study of the capacity of cancer cell extravasion and proliferation in distal tissues to establish metastic growth. Data presented in (Figure 3 A, B) using fluorescent intensity as read out for cancer cell proliferation (same for C, D), which is quite a crude way of quantification. I wonder if it is possible to use more precise methods such as EdU incorporation or pH3 staining, ki67 staining or PCNA staining? Perhaps authors could share their experiences of make some comments on other ways of evaluating the cancer cell proliferation in vivo after xenograft.  

 The authors focused on using the zebrafish xenograft models for metastasis analysis. Angiogenesis was one the first assays developed using zebrafish embryo xenograft model ( Nicoli S, et al). As a methodology, the protocol presented here can be adapted for angiogenesis analysis. Perhaps they could comment on how their protocol could be adapted for evaluating angiogenesis in vivo. There are new developments of the zebrafish embryo xenograft model for angiogenesis such as ( Britto DD, et al) perhaps they could refer to this work in the introduction or discussion, so as to guide the reader to other and more specific examples.

**Are a suitable application and appropriate end-users identified?**

 Yes

**If applicable, is the statistical analysis and its interpretation appropriate?**

 Partly

**Are the 3Rs implications of the work described accurately?**

 Yes

Is the rationale for developing the new method (or application) clearly explained?

Yes

Is the description of the method technically sound?

Yes

Are the conclusions about the method and its performance adequately supported by the findings presented in the article?

Yes

If any results are presented, are all the source data underlying the results available to ensure full reproducibility?

Yes

Are sufficient details provided to allow replication of the method development and its use by others?

Partly

Reviewer Expertise:

NA

I confirm that I have read this submission and believe that I have an appropriate level of expertise to confirm that it is of an acceptable scientific standard.

# Response

**Published:** 2018-11-29

**Authors:** David Hill

**Affiliations:** Newcastle University, UK, UK

The authors would like to thanks Dr Feng for her insightful comments and suggestions. We detail below the additions and changes we have made specifically in response to this review:

1. We have incorporated all changes as suggested including: catalogue numbers of critical reagents and reference to angiogenesis; and mention of alternative post-mortem analyses for cell proliferation.
2. Whilst we have only observed local movement of tracer beads, we have emphasised the importance of this quality control in the main manuscript.
3. For these studies we chose to analyse movements in a single plane and were able to find significant differences. However, with increased imaging time and use of lenses with limited depth of field, cells can be tracked in three dimensions. Caution should be employed as the light exposure in obtaining such image stacks may affect cell behaviour.
4. The capability of cancer cells to invade blood vessels was cell line dependent, for example, more than 80% of embryos injected with A375 cells had cancer cells within the blood vessels by 72 hours, while C8161 and WM164 cells invaded blood vessels less frequently and also showed variable local movement. These preliminary studies indicate the utility of the zebrafish at pre-regulated embryonic stages to study key aspects of metastatic cancer spread.

# Peer review: Reviewer response for version 1

**DOI:** 10.5256/f1000research.18207.r39771

**Published:** 2018-11-06

**Authors:** Adam Hurlstone

**Affiliations:** 1 Division of Cancer Studies, School of Medical Sciences, Faculty of Biology, Medicine and Health, The University of Manchester, Manchester, UK

I enjoyed reading this methodology paper which lays out clearly steps required to achieve successful xenotransplantation in zebrafish embryos and how subsequent growth and dissemination of cancer cells can be monitored.

 The attached method protocol would be improved by inclusion of the below details:

- Explain how to remove embryos from agarose using forceps
- Indicate an appropriate model of microtitre plate for imaging purposes with an inverted microscope (does imaging require glass bottom plates or a certain grade of plasticware?)
- Specify where they obtained the plastic pin mold: have they manufactured it, requested it, or purchased it?
- Comment on whether 72 h incubation in anesthetic is detrimental to embryo health/development
- Which image analysis modules/tools were used in Velocity and Image J. Mention whether default parameters were selected or otherwise? Why 2D rather than 3D distances were calculated using Velocity? Velocity is relatively expensive proprietary software and may not therefore be widely accessible, whereas Image J is free. Could the whole analysis not be undertaken with Image J?
- Specify an appropriate method of ensuring destruction of the embryos within 120 hpf

 Turning to the rest of the manuscript:

- Explain what metrics would be captured by the analysis depicted in Fig 1B and present a representative graph. Mean/median migration distance? Is there a way of distinguishing between several small clusters of cells or a few larger ones?
- Specify the cell line used for Fig 2. How efficient is the model for capturing intravasation events? How many cells are captured intravasating per hour per embryo?
- The presentation and analysis of data in Fig 3B is inappropriate as these are not independent populations of cells. A line graph and linear regression is the appropriate analysis. Does proliferation contribute to the expansion of cells in the caudal hematopoietic tissue?
- It would be of value to include a statement describing the distribution of fluospheres injected either into the yolk or into the duct of cuvier

**Are a suitable application and appropriate end-users identified?**

 Yes

**If applicable, is the statistical analysis and its interpretation appropriate?**

 Partly

**Are the 3Rs implications of the work described accurately?**

 Yes

Is the rationale for developing the new method (or application) clearly explained?

Yes

Is the description of the method technically sound?

Partly

Are the conclusions about the method and its performance adequately supported by the findings presented in the article?

Yes

If any results are presented, are all the source data underlying the results available to ensure full reproducibility?

Yes

Are sufficient details provided to allow replication of the method development and its use by others?

Partly

Reviewer Expertise:

NA

I confirm that I have read this submission and believe that I have an appropriate level of expertise to confirm that it is of an acceptable scientific standard.

# Response

**Published:** 2018-11-29

**Authors:** David Hill

**Affiliations:** Newcastle University, UK, UK

The authors would like to thanks Dr Hurlstone for his thorough and inciteful review of our article.

1. We have made suggested changes to clarify methodology and correct typographical errors in our article, including details of consumables and of pin-mould manufacture. Importantly, applying forceps to break the agarose away from the embryo allows its release into surrounding media. Toxicity of tricaine was not observed in our studies, but if there are concerns it should be excluded with a specific experimental control. Embryos were killed using a schedule 1 method (destruction of the brain). However, for post-mortem histological analysis, cooling and fixation in 4% PFA was also used.
2. Whilst we used commercially available software, we also recommend use of the Fiji implementation of imageJ ( https://fiji.sc/), which contains tools for measurement and tracking.
3. The cell line used for Figure 2 was the parental A375 human melanoma cell line. We have modified the figure legend to reflect this.
4. We have updated our analysis of Figure 3B to reflect that the same cell populations are measured at two time points by using a paired t-test rather than a student’s t-test, and have updated Figure 3 and the legend for Figure 3 accordingly.
5. We have also included a reference (Verykiou *et al.*, 2018) in the main text. We have used this method of analysis to measure the distance invaded by MEKi-resistant A375 melanoma cells. The use of nuclear-localised fluorescent proteins allow individual cells within a cluster to be distinguished, while the use of membrane dyes are ideal for analysis of primary tumour cells, low-number tumour subpopulations and transient events such as intra/extravasation or interaction of tumour cells with host cells and stroma.
